# Fluoxetine and Paroxetine Exhibit a Protective Effect Against Total Joint Arthroplasty in Patients With Osteoarthritis

**DOI:** 10.7759/cureus.92722

**Published:** 2025-09-19

**Authors:** Andrew M Miner, Gaurav Singh, Haad Arif, Parke Hudson

**Affiliations:** 1 Medicine and Surgery, University of California, Riverside School of Medicine, Riverside, USA; 2 Orthopedics, University of Texas at Tyler, School of Medicine, Tyler, USA; 3 Orthopedic Surgery, Loma Linda University Medical Center, Loma Linda, USA

**Keywords:** joints, orthopedics, osteoarthritis, ssri, total joint arthroplasty

## Abstract

Background

Recent research has elucidated the biochemical mechanism by which some selective serotonin reuptake inhibitor (SSRI) drugs, fluoxetine and paroxetine, slow osteoarthritis (OA) disease progression. This novel study aimed to evaluate the impact of fluoxetine and paroxetine on the risk of undergoing primary total hip (THA) or knee arthroplasty (TKA) among OA patients.

Methods

A retrospective cohort study using the TriNetX network compared OA patients prescribed fluoxetine (n=2478) or paroxetine (n=1500) to matched controls without SSRI exposure. Outcomes were assessed via risk analysis, Kaplan-Meier survival analysis, and hazard ratios (HR).

Results

The absolute risk of needing THA or TKA was significantly lower in both SSRI cohorts compared to matched controls. In the fluoxetine cohort, the relative risk (RR) of undergoing THA or TKA was 0.678 (95% CI: 0.645-0.713), while in the paroxetine cohort, it was 0.791 (95% CI: 0.740-0.845). The fluoxetine cohort had an 8.22% chance of requiring total joint arthroplasty (TJA), compared to 14.15% in controls. The paroxetine cohort had an 8.14% chance versus 8.46% in controls. This corresponds to a 1.1% absolute reduction in risk for fluoxetine (95% CI: 1.0-1.3%, p<0.0001) and a 0.7% reduction for paroxetine (95% CI: 0.5-0.9%, p<0.0001). The fluoxetine cohort had a hazard ratio (HR) of 0.675 (95% CI: 0.642-0.711, p<0.0001), while the paroxetine cohort had an HR of 0.742 (95% CI: 0.694-0.794, p<0.0001).

Conclusion

Patients prescribed either paroxetine or fluoxetine had a statistically significant decrease in risk of undergoing THA or TKA; however, further research is needed to explore other clinical outcomes and therapeutic benefits of SSRIs in OA management.

## Introduction

This research was presented as a poster at both the Orthopedic Research Society annual meeting from February 7-11, 2025, in Phoenix, Arizona, as well as the Western Orthopedic Association annual meeting in Kauai, Hawaii, from July 30-August 2, 2025.

Background

Osteoarthritis (OA) is a degenerative disease of synovial joints, most commonly in the knee, hip, and hand, that affects approximately 595 million people worldwide [1-2]. Most conservative OA treatments focus on pain relief through physical therapy and pharmacologic treatments such as nonsteroidal anti-inflammatory drugs (NSAIDs), acetaminophen, or corticosteroid injections [3]. When conservative treatments fail, total joint arthroplasty (TJA) such as total hip arthroplasty (THA) and total knee arthroplasty (TKA) may be considered. 

While medical treatments can alleviate symptoms, none effectively halt disease progression, and although many new OA drugs are being actively researched as potential tissue-regenerating agents to slow OA progression, further testing is required before they become available on the market [3]. However, recent studies have elucidated the mechanism by which certain selective serotonin reuptake inhibitors (SSRIs) slow, and even reverse, OA progression by modulating various biochemical signaling pathways involved in inflammation, such as the Wnt/β-catenin and G protein-coupled receptor kinase 2 (GRK2) pathways [3-5]. While this research focuses on specific molecular pathways, the clinical significance of these effects in relation to OA remains unknown. To our knowledge, no previous studies have evaluated their effect on the need for future joint arthroplasty. 

We sought to identify the effect of paroxetine and fluoxetine, two commonly prescribed SSRIs, on the likelihood of future TJA in patients with OA. We hypothesize that patients with OA undergoing concomitant treatment with either paroxetine or fluoxetine were less likely to progress to eventual THA or TKA as opposed to patients with OA who were not taking paroxetine or fluoxetine.

## Materials and methods

Data source

This study queried the TriNetx US collaborative Network on November 30th, 2024, which included 68 de-identified healthcare organizations (HCOs) within the United States, encompassing approximately 118 million patients. The TriNetX database is a nationwide database composed of deidentified patient information that allows for an analysis of patient data such as diagnoses, surgeries, prescriptions, lab values, and even genomic data [6]. 

Ethics statement

Due to the de-identified nature of these data, human subjects approval from an institutional review board (IRB) was not necessary. Instead, the University of California, Riverside IRB has reviewed this project and determined this research to be non-human subjects research.

Study population

Two cohorts and their individually matched control groups were included in this study. The study cohorts consisted of 1) patients aged ≥18 years, 2) diagnosed with OA, and 3) undergoing medical treatment with fluoxetine (cohort one) or paroxetine (cohort two). The control cohort included 1) patients aged ≥18 years, 2) diagnosed with OA, and 3) without any SSRI use.

Exclusion criteria for all cohorts included a concurrent diagnosis of the following OA risk factors: osteonecrosis, osteitis deformans, internal derangement of the knee, acquired limb deformities, congenital hip deformities, achondroplasia, or overweight/obesity. The index event for each cohort was defined as the first healthcare organization (HCO) visit meeting all inclusion criteria. Index events occurring more than 20 years ago were excluded, as were patients who experienced a THA or TKA prior to their index event. Therefore, the final cohorts contained individuals who met all the inclusion criteria between the years 2004-2024. 

**Figure 1:**
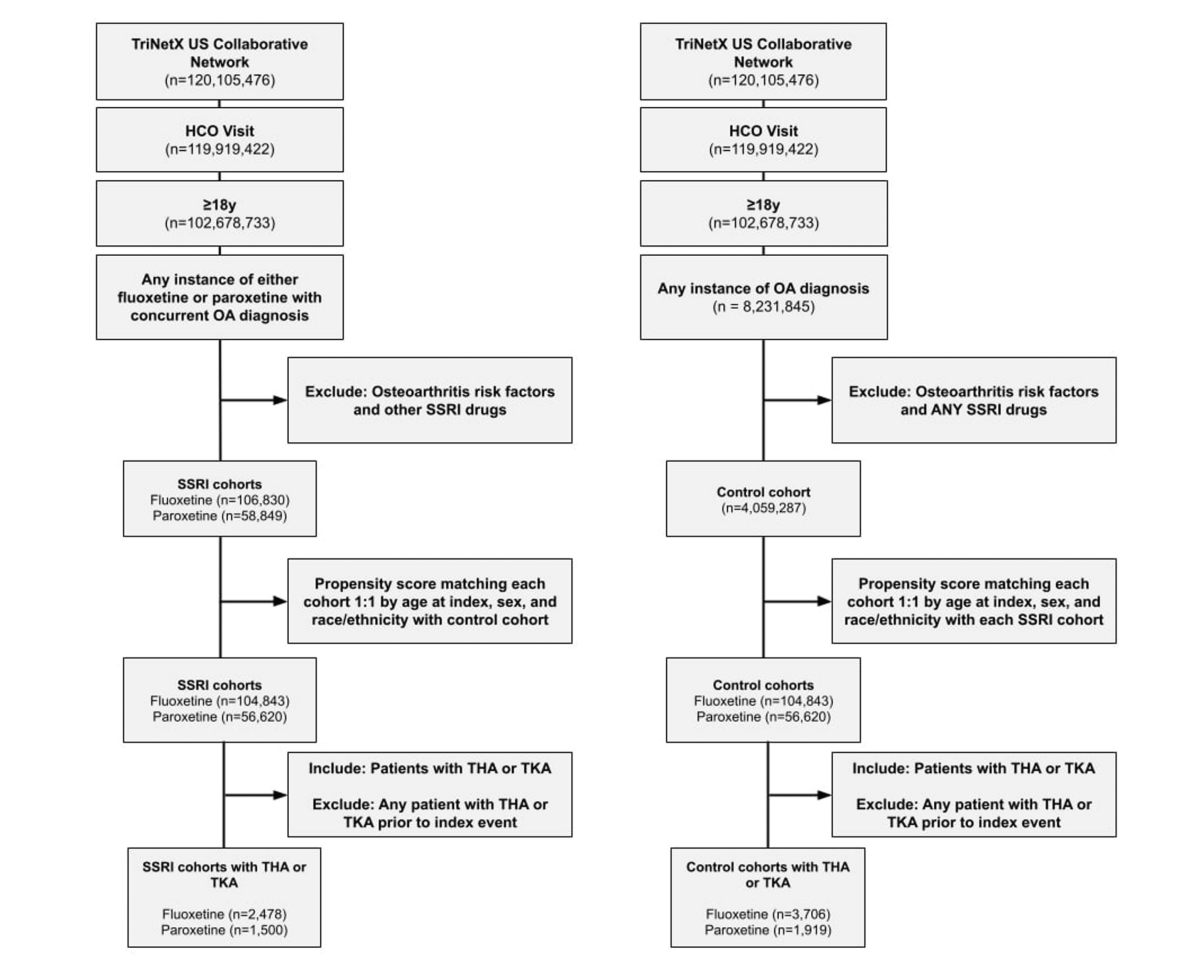
Diagram depicting selection of both SSRI cohorts and their respective control cohorts HCO - healthcare organization; THA - total hip arthroplasty; TKA - total knee arthroplasty; SSRI - selective serotonin reuptake inhibitors

Each SSRI cohort was compared to the control cohort of OA patients without SSRI exposure. Figure 1 outlines the cohort selection process and population sizes at each step.

Statistical analysis

Propensity score matching was performed to balance each study cohort with the control cohort based on age at index event, sex, race, and ethnicity. To make sure the groups we compared were as similar as possible, we used propensity score matching. This is a statistical technique that pairs patients in the treatment group with patients in the control group who have very similar baseline characteristics (like age, sex, comorbidities). The TriNetX system does this automatically using what's called a nearest neighbor method: for each patient in one group, it finds the "closest match" from the other group. Each patient can only be used once, so no one is counted twice. We also required the match to be fairly close: within 0.10 standard deviations of the calculated score that summarizes their baseline risk factors. This helped reduce bias and ensured the groups were well-balanced before comparing outcomes.

Outcomes were compared using risk analysis, a Kaplan-Meier 7300-day (20-year) survival analysis, and hazard ratios (HR). Statistical significance was defined as a p-value <0.05 and a 95% confidence interval (95% CI). These analyses were performed utilizing the TriNetX Analysis platform (TriNetX, LLC, Cambridge, MA). The software reports effect estimates, 95% confidence intervals, and associated p-values; however, the underlying test statistics (e.g., t, χ², F) are not provided by the platform.

## Results

Patient characteristics

Table 1 and Table 2 summarize the baseline demographics of cohort one and cohort two before and after propensity score matching, respectively. The mean age at the index event after matching was performed for both the study and control cohorts was 62.6 ± 14.0 years for cohort one and 66.0 ± 13.3 years for cohort two. Each cohort was matched well to its respective control cohort. 

**Table 1:** Fluoxetine cohort characteristics Std diff - standard difference; SSRI - selective serotonin reuptake inhibitors

Characteristic	Before matching - control (n=3,866,870)	Before matching - SSRI (n=104,843)	Std Diff	After matching - control (n=104,843)	After matching - SSRI (n=104,843)	Std Diff
Age at Index, mean ± SD	64.0 ± 14.4	62.6 ± 14.0	0.101	62.6 ± 14.0	62.6 ± 14.0	<0.001
Gender - female, n (%)	2,036,509 (52.7)	70,727 (67.5)	0.306	70,727 (67.5)	70,727 (67.5)	<0.001
Gender - male, n (%)	1,598,586 (41.3)	30,027 (28.6)	0.269	30,029 (28.6)	30,027 (28.6)	<0.001
Gender - unknown, n (%)	231,775 (6.0)	4089 (3.9)	0.097	4087 (3.9)	4089 (3.9)	<0.001
Race - White, n (%)	2,573,111 (66.5)	79,058 (75.4)	0.196	79,058 (75.4)	79,058 (75.4)	<0.001
Race - Black or African American, n (%)	430,831 (11.1)	7813 (7.5)	0.127	7813 (7.5)	7813 (7.5)	<0.001
Race - Pacific Islander, n (%)	29,539 (0.8)	45 (0.0)	0.114	45 (0.0)	45 (0.0)	<0.001
Race - Asian, n (%)	103,639 (2.7)	1186 (1.1)	0.113	1186 (1.1)	1186 (1.1)	<0.001
Race - American Indian, n (%)	9668 (0.3)	238 (0.2)	0.14	238 (0.2)	238 (0.2)	<0.001
Race - unknown/other, n (%)	720,082 (18.6)	16,503 (15.7)	0.054	16,503 (15.7)	16,503 (15.7)	<0.001

**Table 2:** Paroxetine cohort characteristics Std diff - standard difference; SSRI - selective serotonin reuptake inhibitors

Characteristic	Before matching - control (n=3,866,901)	Before matching - SSRI (n=56,620)	Std Diff	After matching - control (n=104,843)	After matching - SSRI (n=104,843)	Std Diff
Age at index, mean ± SD	64.0 ± 14.4	66.0 ± 13.3	0.146	66.0 ± 13.3	66.0 ± 13.3	<0.001
Gender - female, n (%)	2,036,523 (52.7)	37,940 (67.0)	0.306	37,940 (67.0)	37,940 (67.0)	<0.001
Gender - male, n (%)	1,598,603 (41.3)	16,298 (28.8)	0.269	16,298 (28.8)	16,298 (28.8)	<0.001
Gender - unknown, n (%)	231,775 (6.0)	2382 (4.2)	0.082	2382 (4.2)	2382 (4.2)	<0.001
Race - White, n (%)	2,573,111 (66.5)	42,108 (74.4)	0.172	42,108 (74.4)	42,108 (74.4)	<0.001
Race - Black or African American, n (%)	430,831 (11.1)	4382 (7.7)	0.117	4382 (7.7)	4382 (7.7)	<0.001
Race - Pacific Islander, n (%)	29,539 (0.8)	39 (0.1)	0.108	39 (0.1)	39 (0.1)	0.005
Race - Asian, n (%)	103,639 (2.7)	697 (1.2)	0.105	697 (1.2)	685 (1.2)	0.002
Race - American Indian, n (%)	9668 (0.3)	144 (0.3)	0.001	144 (0.3)	144 (0.3)	<0.001
Race - unknown/other, n (%)	720,113 (18.7)	9250 (16.3)	0.042	9255 (16.3)	9250 (16.3)	<0.001

Outcomes

**Figure 2:**
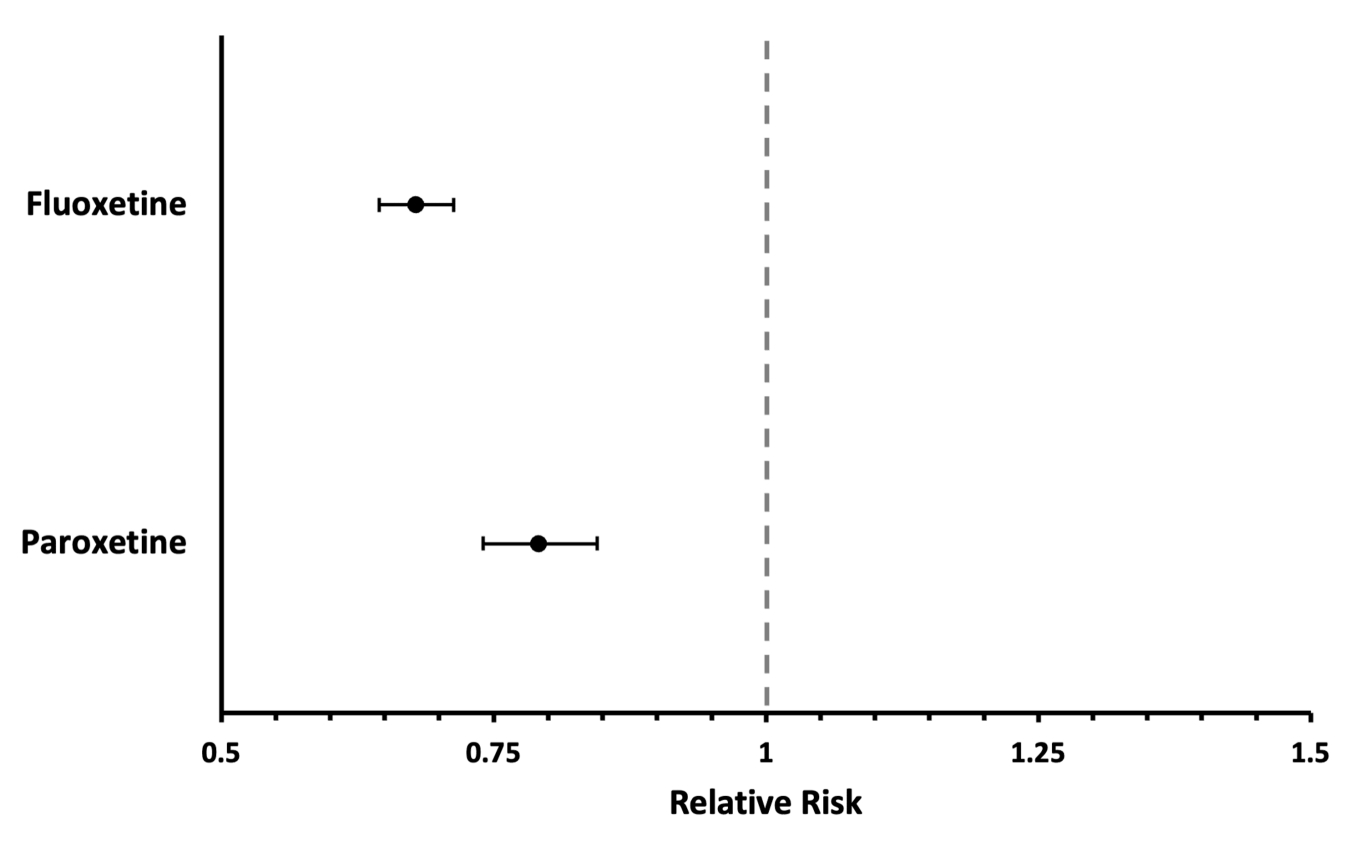
Relative risk of each SSRI cohort undergoing either THA or TKA when compared to propensity score-matched controls THA - total hip arthroplasty; TKA - total knee arthroplasty; SSRI - selective serotonin reuptake inhibitors

The absolute risk of needing hip or knee arthroplasty was significantly lower in both SSRI cohorts compared to their matched control groups. In cohort one, the relative risk (RR) of undergoing THA or TKA was 0.678 (95% CI: 0.645-0.713), while in cohort two the RR was 0.791 (95% CI: 0.740-0.845) (Figure 2). These findings correspond to a 1.1% absolute reduction in risk for cohort one (95% CI: 1.0%-1.3%, p<0.0001) and a 0.7% reduction for cohort two (95% CI: 0.5%-0.9%, p<0.0001). These findings are shown in Table 3.

**Figure 3:**
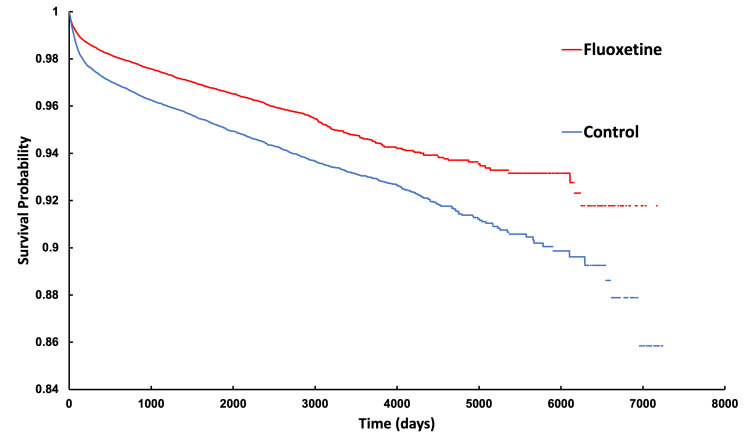
Kaplan-Meier analysis of fluoxetine and control cohorts indicating probability of not having undergone THA or TKA as a function of time THA - total hip arthroplasty; TKA - total knee arthroplasty

**Figure 4:**
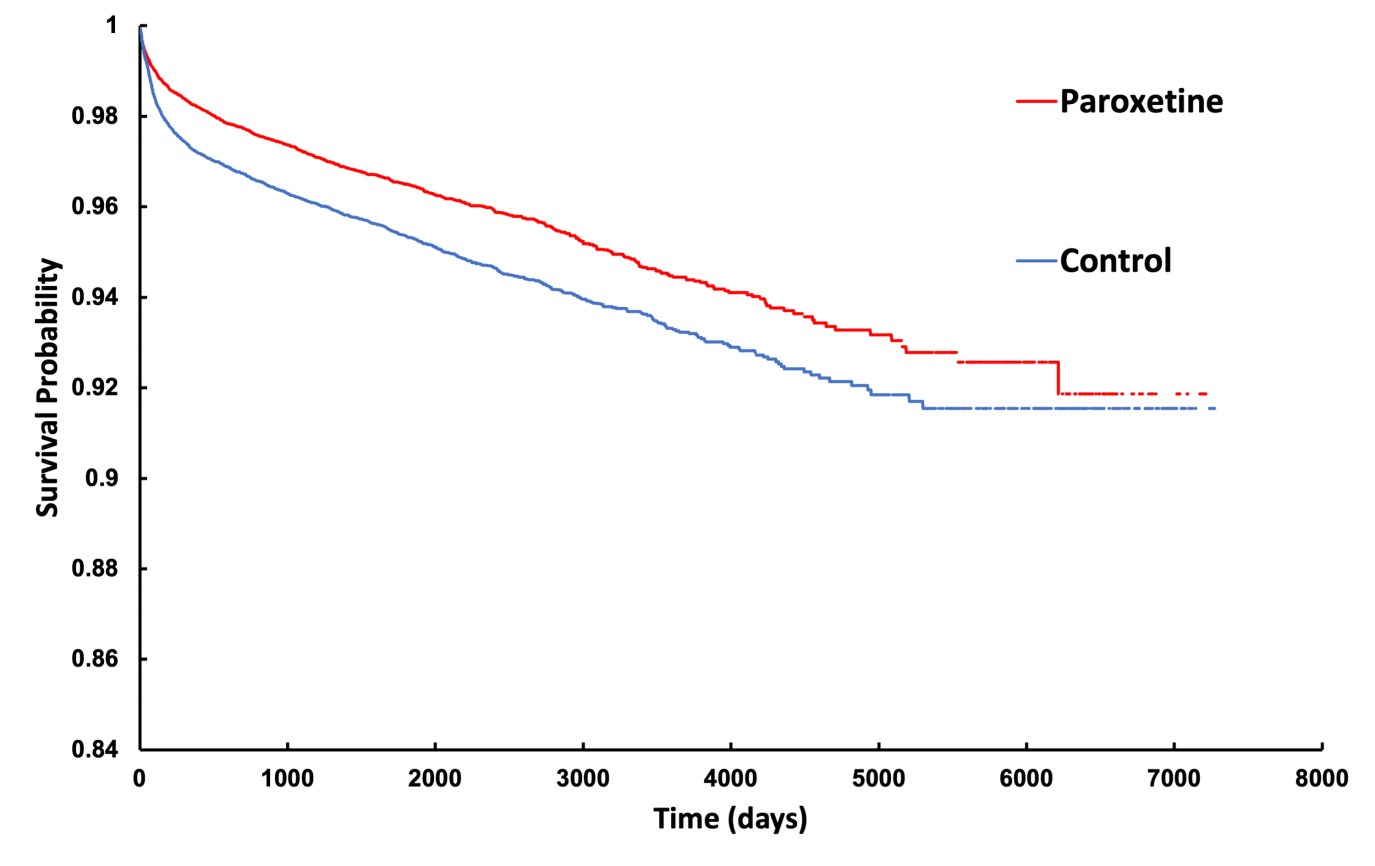
Kaplan-Meier analysis of paroxetine and control cohort indicating probability of not having undergone THA or TKA as a function of time THA - total hip arthroplasty; TKA - total knee arthroplasty

Kaplan-Meier survival curves (Figures 3, 4) further illustrate these differences. At the end of the 7300-day study period, patients in the fluoxetine cohort had an 8.22% chance of requiring a TJA compared to 14.15% in their matched control group. Similarly, patients in the paroxetine cohort demonstrated an 8.14% chance of requiring TJA compared to 8.46% in their matched control group. Both analyses demonstrated statistically significant results, with log-rank test p-values<0.0001. Additionally, cohort one exhibited a hazard ratio of 0.675 (95% CI: 0.642-0.711, p<0.0001) while cohort two had a HR of 0.742 (95% CI: 0.694-0.794, p<0.0001). These findings indicate a lower likelihood of requiring TJA over time for patients in the SSRI cohorts compared to their matched controls; however, the gaps in the curves represent short periods of time in which follow-up data were not reported in TriNetX. For binary outcomes, Cohen's d effect sizes were estimated from the calculated risk ratios using the log-transformation conversion formula described by Chinn (2000) [7], which yielded a result of -0.214 for the fluoxetine cohort and -0.129 for the paroxetine cohort, further demonstrating lower TJA rates in these cohorts.

**Table 3:** Risk and odds ratios in fluoxetine and paroxetine cohorts CI - confidence interval; SSRI - selective serotonin reuptake inhibitors

Cohort	Patients in cohort	Patients with outcome	Risk	Risk difference	Risk ratio (95% CI)	Odds ratio (95% CI)
Fluoxetine - control	104,878	3,706	0.036	-0.011 (-0.013, -0.010)	0.678 (0.645, 0.713)	0.670 (0.636, 0.705)
Fluoxetine - SSRI only	102,787	2,478	0.024	-0.011 (-0.013, -0.010)	0.678 (0.645, 0.713)	0.670 (0.636, 0.705)
Paroxetine - control	56,270	1,919	0.034	-0.007 (-0.009, -0.005)	0.791 (0.740, 0.845)	0.785 (0.733, 0.841)
Paroxetine - SSRI only	55,613	1,500	0.027	-0.007 (-0.009, -0.005)	0.791 (0.740, 0.845)	0.785 (0.733, 0.841)

## Discussion

Key results

Our findings show that SSRIs may offer a modest protective effect in reducing the need for TKA and THA with OA-related joint pain. In terms of relative risk reduction, fluoxetine demonstrated a 32.2% reduction in THA and TKA outcomes compared to the control group. For paroxetine, the risk ratio reflected a 20.9% reduction in risk compared to the control group. The odds ratios also supported these findings, with fluoxetine showing an odds ratio (OR) of 0.670 (95% CI: 0.636-0.705) and paroxetine an OR of 0.785 (95% CI: 0.733-0.841), both indicating a minor reduction in the odds of undergoing arthroplasty with treatment. These findings suggest that SSRIs may help delay or prevent the need for hip or knee arthroplasty, procedures associated with significant risks and morbidity [8].

While both THA and TKA are considered effective interventions for improving the quality of life for OA patients, both procedures carry the potential for complications, including serious and minor adverse events. Serious complications such as pulmonary embolism, deep vein thrombosis, cardiac events, and joint infections can result in extended hospital stays or the need for reoperation. Additionally, both procedures are associated with a 30-day readmission rate of roughly 3%, further compounding patient morbidity [9-10]. These potential complications demonstrate the risks and burden of TJA, underscoring the potential benefits of non-surgical interventions. SSRIs could provide a useful adjunct to current OA treatments, offering a way to mitigate the need for surgery and reduce patient exposure to surgical risks [1,3,8,11]. 

The growing demand for THA and TKA, driven by an aging population, highlights the need for improved, non-surgical interventions to effectively manage OA symptoms and reduce or delay the need for surgical intervention [2]. However, the clinical significance of the risk reduction observed in our study must be considered carefully, as it may not lead to large-scale changes in treatment approaches [9-10]. Patient selection is a critical consideration when considering the initiation of concomitant SSRI treatment, as SSRIs have been shown to reduce bone mineral density, making it particularly important among the elderly population. SSRIs may be most attractive in patients with coexisting OA and major depressive disorder (MDD) or generalized anxiety disorder (GAD). In situations where patients do ultimately require TJA, SSRI use has been associated with a reduced risk of THA or TKA revisions [12]. 

Going forward, it is essential to prioritize further research aimed at developing preventative strategies for THA and TKA. SSRIs may have a place in the pharmacological arsenal, yet it still remains unclear if they can serve as a practical adjunct to traditional OA management strategies [3,11,13]. 

Limitations

While the benefits of using TriNetX include access to over 100 million patients, it is encumbered by many of the same limitations as other large, national database studies, such as a lack of granularity in the data provided. Patient compliance with SSRI medications was not available due to the nature of the study. The investigation's findings are also notably constrained by the absence of data regarding the duration and dosage of SSRI usage. This limitation diminishes the ability to draw significant conclusions about the length of time and the SSRI therapeutic dose required to decrease TKA and THA rates. Stages of OA severity were also not available in the TriNetX patient data pool, thus limiting our ability to draw further conclusions through a stratified analysis. Additionally, this study may help clarify the relationship between SSRI therapy and its potential for concomitant use. This is particularly relevant considering that adherence to antidepressant medication regimens tends to diminish over time [14]. However, treatment of MDD or GAD with SSRI drugs may also be a potential confounder in and of itself. While these drugs do exhibit biological effects that attenuate OA progression, improvement of depressive symptoms may also lead to patients avoiding future need for TJA via increased motivation to assume better lifestyle habits, such as improved diet and increased exercise, both of which have been associated with reduced inflammation and compressive knee forces [15]. Therefore, a randomized clinical trial examining the duration and dosage of SSRIs could yield more nuanced insights into the effectiveness of treatment in preventing the progression of OA to TKA and THA.

## Conclusions

This study demonstrates that patients with osteoarthritis prescribed fluoxetine or paroxetine had a modest but statistically significant reduction in the risk of undergoing total hip or knee arthroplasty compared with matched controls. While the absolute risk reduction was small, the relative decrease observed, particularly with fluoxetine, suggests that SSRIs may offer protective effects against OA progression. These findings align with emerging evidence that SSRIs modulate inflammatory pathways implicated in OA, thereby offering a potential therapeutic benefit beyond their psychiatric indications.

However, the clinical implications of this risk reduction should be interpreted with caution, given the limitations inherent in retrospective database studies, including the lack of information on medication adherence, dose, and OA severity. Nevertheless, our results highlight an important and previously underexplored association between SSRI use and OA outcomes. Future prospective and randomized studies are warranted to validate these findings, clarify the optimal context for SSRI use in OA management, and determine whether these agents can be integrated into broader non-surgical strategies to delay or prevent joint replacement, especially in patients with concomitant mental health conditions necessitating SSRI use. 
